# How the self-concept structures social role learning: insights from computational models

**DOI:** 10.1098/rsos.250590

**Published:** 2025-09-24

**Authors:** Josue Garcia-Arch, Marc Sabio-Albert, Christoph W. Korn, Lluis Fuentemilla

**Affiliations:** ^1^ Department of Cognition, Development and Education Psychology, University of Barcelona, Barcelona, Spain; ^2^ Institute of Neuroscience, Barcelona, Spain; ^3^ Heidelberg University, Heidelberg, Baden-Württemberg, Germany; ^4^ University of Barcelona, Barcelona, Spain; ^5^ Bellvitge Institute for Biomedical Research, Barcelona, Spain

**Keywords:** social learning, computational models, reinforcement learning, self-concept clarity, social cognition

## Abstract

Learning about the social expectations tied to upcoming social roles is crucial to promoting adaptation. However, such learning can prompt a strong need for personal change, undermining the stability of individuals’ self-concept. Here, we provide a mechanistic account of how individuals at the onset of significant life transitions utilize their self-concept to modulate self-role dissonances during social role learning. Participants engaged in a learning task where they first provided self-ratings for different traits and then estimated how these traits would apply to an individual well-adapted to their forthcoming social role and received trial-by-trial feedback from reference groups. We hypothesized that individuals would employ strategies to minimize dissonances between role expectations and their current self-concept during the learning process. Our computational models included strategies that straightforwardly integrate role expectations to more complex strategies that involve leveraging the self-concept against the pure incorporation of role-related information. The best-performing model demonstrated that the self-concept functions as a modulatory mechanism, guiding the integration of role information to avoid self-role dissonances. Notably, this strategy was strongly accentuated in individuals learning about their upcoming contexts. Our work offers a mechanistic perspective on role learning that may inform interventions to support those facing significant life transitions.

## 1. Introduction

Stability and change are essential components of human development. In the absence of pressing environmental demands, we tend to develop a clear and stable self-concept [1,2], which is crucial for psychological well-being [1,3]. However, societal pressures often compel us to adapt to new realities. Consider, for example, the challenge experienced by adolescents’ self-concept as they transition into young adulthood and confront novel academic and social responsibilities. Events like career shifts, becoming a parent or relocating to a different cultural environment exemplify situations necessitating a delicate balance between self-consistency and the recognition of necessary changes driven by societal expectations. The anticipation of these transitions pressures individuals to learn about the characteristics and behaviours deemed adaptive within a new social setting [4–7]. However, learning about a new role can signal a strong need for personal change, potentially destabilizing our self-concept and creating a paradox where both stability and adaptation are essential. Despite the relevance of understanding these dynamics, the mechanisms by which individuals learn about the necessary characteristics for successful adaptation to their upcoming social roles remain unexplored.

The importance of maintaining a clear and stable self-concept is well-established across different domains of psychological functioning and well-being. For example, a stable self-concept contributes to better social relationships, better mental health, psychological adjustment, self-esteem and greater resilience against adverse life events [1,3,8–10]. However, transitions to new social roles threaten this stability, requiring individuals to adapt to new demands and redefine their self-views [11]. Despite the potential challenge to self-concept stability, identifying with a new role is crucial for establishing a sense of belonging within a social group and gaining the support of others who share the same identification [12–14]. In turn, a lack of identification with one’s social role has been related to psychological distress [15]. Current evidence from qualitative and correlational research suggests the learning processes underlying the identification with new social roles might require a trade-off between preserving self-concept integrity and anticipating the necessary shifts to meet evolving social demands [11,14,16–18].

According to existing evidence, individuals learning about forthcoming social roles will face a dual need, namely, to map which traits and behaviours are more likely to provide adaptation to the future social environment and avoid the maximization of dissonances between these social expectations and their current self-concept. Here, self-role dissonances could be conceptualized as error-like signals calling for an adaptation that might be difficult to implement for the individual due to behaviour and self-concept’s tendency towards stability [1,3,7,19–22]. Indeed, the negative effects of perceiving tension between current and required self-states have been well-established [7,23–25]. In this sense, individuals learning about their forthcoming social roles might display a biased sensitivity against social cues that signal the need for self-concept adaptation [19], or they might use their self-concepts as reference points to force the alignment of role-related information with their current self-views [7,26]. To gain a deeper understanding, research needs to incorporate methodologies able to capture the complexities involved in this learning process. Computational modelling offers a powerful alternative capable of providing a granular and mechanistic understanding of the dynamics involved in role learning.

Computational models have proven to be useful for understanding different social learning processes [7,27–31]. For example, studies have shown that these models can account for how individuals learn about specific characteristics, behaviours or emotional states of other individuals [30,32–36]. Importantly, recent research has suggested that these models can also be used to explore how individuals learn about more complex constructs, such as multidimensional representations of others’ personalities. These studies unveiled that learning about the multifaceted representations of others’ personalities involves leveraging the intricate web of relationships among the characteristics being learned [37,38]. Moreover, individuals learning about the profiles of individual persons make use of general social knowledge about group memberships. Such insights suggest that learning about complex social constructs such as social roles is not isolated to individual characteristics but is influenced by the interconnectedness of its elements (e.g. personality traits), guiding individuals in constructing a coherent understanding. However, the dynamics involved in role learning are likely to extend beyond pure knowledge acquisition, as they have the potential to impact an individual’s identity. In turn, the self-concept is a crucial framework for interpreting and organizing internal and external representations of reality [39]. Therefore, computational models of role learning should incorporate mechanisms that consider the self-concept as a latent structure capable of regulating self-role dissonances during the learning process.

We introduce a computational approach to formalize the trade-off between learning the social expectations tied to upcoming social roles and maintaining self-concept stability. We frame the problem as a learning process in which two simultaneous needs must be reconciled. The first need is to be accurate about which traits might maximize future successful adaptation by integrating social expectations about which traits should be ideally expressed in an upcoming role to adapt to the reward structure of the anticipated environment. The second need, consistent with motivated reasoning [40], is to minimize disruptions to the individual’s self-concept by avoiding the maximization of signals that point towards a strong need to change [7,41], i.e. minimize self-role dissonances during the learning process. To examine this possibility, we formalized a series of computational models reflecting distinct learning strategies. These models span from strategies that straightforwardly integrate social expectations about new roles to more complex strategies that involve leveraging the current self-concept against the pure incorporation of role-related information.

We conducted two studies to elucidate the computational mechanisms involved in role learning. Our studies targeted two distinct populations at the onset (or right before) of a new role acquisition that learned about the abstract, general representations of well-adapted exemplars of their forthcoming social role. The first population was composed of first-year undergraduate students recruited during the first semester of the academic course. This setting offered a context for exploring how emerging adults experiencing a life transition [42] learn about their new role as college students. The second cohort consisted of pregnant women, a population at the onset of a profound social role transition [43,44]. This allowed us to probe the mechanisms of role learning in a context marked by important changes in responsibilities and societal pressures and test the generalizability of the learning dynamics under study.

## 2. Experiment 1

### 2.1. Methods

#### 2.1.1. Participants

Inclusion criteria common for the two experiments reported in this study included (i) age equal to or above 18 years old and (ii) no self-reported history of psychiatric disorders. Similar to prior research [37,45], exclusion criteria common to all studies included (i) missing more than 20% of the trials during the experimental task and (ii) giving the exact same rating more than 80% of the time.

We recruited a sample of 130 participants through the lab panel of the University of Barcelona. This sample size was based on prior studies with similar analytical strategies [37,38]. The experiment included two groups that learned about either a self-relevant (‘well-adapted psychology student’) or a non-self-relevant (‘well-adapted police officer’) role, with participants randomly assigned to each condition. The self-relevant role group consisted of 65 participants (54 females) with an average age of 18.58 (s.d. = 0.99). Two participants were excluded because of missing more than 20% of the trials, and one participant was excluded due to giving the exact same rating during the whole task. The final sample was composed of 62 individuals (53 females). The non-self-relevant role group included 65 participants (51 females) with an average age of 18.93 (s.d. = 1.56). One participant was excluded because of giving the exact same rating more than 80% of the time. For this group, we also asked participants whether there was any chance they might consider becoming a police officer (the role they would learn about) in the future. We used this question as an additional exclusion criterion to ensure participants would not learn about a role they might consider pursuing. Four participants responded positively to the question and were excluded from further analysis. The final sample was composed of 59 individuals (49 females). All studies reported in this manuscript took place between January and March 2024 and were approved by the local research ethics committee (University of Barcelona’s Bioethics Commission: IRB00003099). Studies were not pre-registered.

#### 2.1.2. Procedure

**Figure 1.**
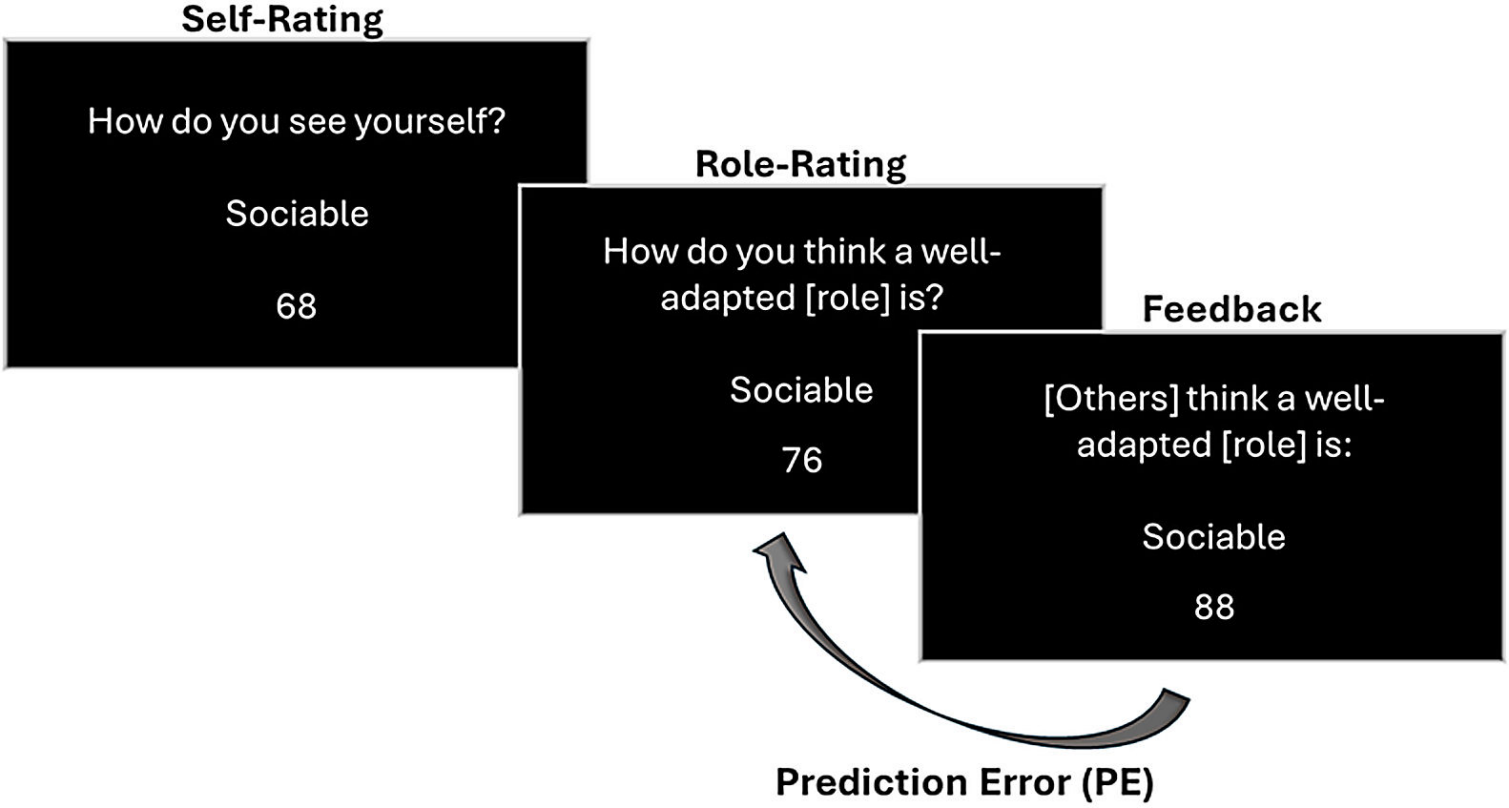
Overview of the experimental task. Participants first provided a self-rating for a given trait (e.g. ‘Sociable’) on a scale from 1 (‘not at all’) to 100 (‘extremely’). Next, they evaluated how well they thought the trait applied to individuals well-adapted to a specific role (e.g. ‘psychology student’) and received feedback showing the average rating for that trait by a reference group (others) (i.e. senior psychology undergraduates, experienced mothers or a diverse cohort of individuals). The difference between the participant’s role rating and the feedback score represents the PE. Judgements were separated by inter-trial intervals of 500 ms. This process was iterated for a set of 50 different traits (randomly distributed).

Participants conducted a social learning task in which they provided both self-ratings and their estimations of role-specific attributes and received feedback from reference groups (figure 1). At the beginning of each trial, participants encountered the prompt ‘How do you see yourself?’ accompanied by an adjective (e.g. ‘Sociable’). Below this prompt, a slider scale ranging from 1 to 100 was provided for participants to rate how much they believed the trait applied to them, with 1 indicating ‘not at all’ and 100 meaning ‘extremely’. After providing their self-rating, participants were asked to provide an evaluation for the same trait in relation to the predefined role they were learning about, answering the question, ‘How do you think a well-adapted [role] (e.g. ‘psychology student’) is?’. Participants were instructed that by ‘well-adapted’ we referred to an individual who successfully meets the typical demands and expectations associated with the role. Specifically, they were asked to consider a person whose traits and behaviours effectively promote optimal performance, adjustment and positive outcomes in the context of that particular role. They had 15 s to provide each rating. Directly after this estimate, participants received feedback consisting of the average estimates of reference groups (e.g. senior psychology undergraduates) on the role ratings. The feedback appeared on the screen in the form ‘[e.g. 4th grade psychology students] think a well-adapted [e.g. psychology student] is:’ followed by the same adjective participants just evaluated and a score ranging from 1 to 100. This score was displayed on the screen for 3 s. This sequence was repeated for the 50 traits included in the experimental task. The order of traits was randomized across participants. Importantly, participants were not instructed to learn during the task.

#### 2.1.3. Stimuli

Following prior related research [7,19,21,37,46,47], participants learned about trait adjectives such as ‘Sociable’ or ‘Anxious’. Stimuli consisted of 25 positive and 25 negative traits selected from prior studies [7,19,22,37,47], which come from widely studied lists of personality descriptors [48] (electronic supplementary material, Table S1). Adjectives represented a broad spectrum of personality attributes, such as those included in the HEXACO model of personality [49] (e.g. Honesty–Humility: ‘Honest’, Emotionality: ‘Anxious’, Extraversion: ‘Sociable’, Agreeableness: ‘Tolerant’, Conscientiousness: ‘Disorganized’, Openness: ‘Original’) together with trait adjectives representing additional dimensions (e.g. ‘Authoritarian’, ‘Practical’). Similar to prior research [19,22,28,46,47], we chose trait adjectives as indicators of self-concept units because they concisely represent stable, generalized self-knowledge accumulated through multiple life experiences and interactions [2,50–52]. Hence, these descriptors provide robust, theoretically grounded and empirically supported representations of individuals’ self-conceptualizations.

### 2.2. Computational models

We tested five computational models to explore which model best described participants’ learning strategies. Our models were inspired by recent research on learning about others’ personalities [37]. This research indicates that when learning about others, participants use fine-grained inter-trait relationships to spread prediction errors (PEs) and promote learning. This learning mechanism (henceforth, fine granularity) entails the adjustment of expectations for upcoming role attributes based on the discrepancy between the participant’s estimation of a given trait (e.g. ‘sociable’) and the feedback received (that is, PE) via a similarity matrix (SIM; electronic supplementary material, figure S4). That is, if a participant has a PE of ‘30’ for the trait ‘Sociable’, this PE will be spread to the rest of the upcoming traits in the task according to their correlation. For example, imagine that the trait ‘Friendly’ has a correlation with ‘Sociable’ of 0.5. In this situation, the trait ‘Friendly’ will be updated by the PE received for the trait ‘Sociable’ as PE_sociable_ × 0.5 (*r*_(sociable, friendly)_). In turn, this update will be modulated by the learning rate, which is estimated as a free parameter for each participant, determining their sensitivity to PEs. SIMs (Pearson correlations), along with feedback ratings, were computed from the ratings provided by separate, external groups of individuals (electronic supplementary material, figure S4). Four of our five computational models were operationalized as hybrid reinforcement learning models, including, but not limited to, a fine granularity learning mechanism. The remaining model consisted of a simple regression that assumes role attribute estimations derived directly from a linear transformation of self-ratings, representing ‘no learning’. Models 2–5 make use of the PE to update the prediction (P) for the following trials. The PE for all models is the feedback (F) on a certain trial (t) minus the prediction (P) on that trial. Initial feedback expectations (E) were defined by treating the first trait’s expectation as a free parameter [37], and subsequent expectations were adjusted via linear regressions based on the inter-trait SIM (electronic supplementary material, figure S4). All ratings related to negative adjectives were reverse-coded. Next, we describe all models included in detail.

#### 2.2.1. Model 1: no learning

Model 1 assumes that participants perform a linear transformation of their self-ratings (S) to predict (P) role ratings. This model performs like a standard linear regression where *β*_0_ represents the intercept and *β*_1_ the slope.


$$P=\beta_{0}\;+\beta_{1}\cdot S.$$

#### 2.2.2. Model 2: fine granularity

Model 2 employs fine-grained granularity and updates all upcoming traits in each trial based on how similar they are to the current item. That is, on a trial-by-trial basis, model 2 updates the estimates of upcoming traits based on the current PE and the learning rate. Moreover, it weights the spread of the PE to upcoming trials by means of a SIM. This interconnected updating aligns with prior theoretical and computational research proposing that knowledge structures, particularly trait-based representations, are embedded within semantic networks that facilitate generalized learning from feedback [7,28,29,37,38]. This involves that on each trial, the PE for the current trait affects upcoming traits as a function of how similar upcoming traits are to the trait being evaluated. Here, *E*(*t*) stands for the initial feedback expectation for the current trial *t*, *α* for the learning rate and SIM for the similarity matrix.


$$P\left(t\;+1\right)=E\left(t\right)+\sum_{i=2}^{t-1}\alpha \;\cdot PE(i)\;\cdot SIM(i,t+1).$$

#### 2.2.3. Model 3: fine granularity (2 learning rates)

Model 3 extends model 2 by including asymmetric learning dynamics with two distinct learning rates. One learning rate {+} is applied when the current feedback (*F*) reduces the dissonance between self-ratings and participants’ estimation of role ratings (|*F* − *S*| < |*P* − *S*|). The other learning rate {−} is applied in the opposite case, i.e. when |*F* − *S*| > |*P* − *S*|. This model captures differential sensitivity to PEs that either amplify or minimize self-role dissonances. That is, differential integration of that feedback is capable of enhancing or disrupting the stability of individuals’ self-concept [7,41]. Asymmetric updating has been most often related to valenced feedback (whether binary outcomes or scalar differences [47,53–55]), and recent literature indicates that individuals also show asymmetric updating based on whether feedback reinforces or challenges the stability and coherence of their self-views [19,28,56].


$$P(t+1)=E(t)+\sum_{i=2}^{t-1}\alpha \left\{+,-\right\}\cdot \;PE(i)\;\cdot SIM(i,t+1).$$

#### 2.2.4. Model 4: self-adjusted fine granularity

Model 4 extends model 2 by including self-ratings into model’s equation. It operates by combining the self-ratings with the predictions derived from fine granularity learning, employing a balancing factor to weigh the contribution of self-ratings against the learning-based predictions for each trial, shrinking role estimations towards self-ratings. Moreover, in contrast to model 3, which differentiates between sensitivity to feedback that amplifies or reduces self-role dissonance, model 4 pulls learned role estimations towards the self-concept itself, using a participant-specific weighting. This mechanism consistently regulates the influence of feedback, preventing the maximization of self-role dissonances when feedback diverges from the self and facilitating convergence when feedback narrows existing gaps. This approach reflects the long-standing idea that the self-concept serves as a psychological anchor, ensuring stability and coherence across diverse learning situations [2,7,19,39,57]. Information resulting from the fine granularity learning is balanced by the self-ratings by means of the weighting parameter *γ* (bounded (0 1)). This parameter determines how much participants rely on just the learning mechanism from model 2 or their current self-concept. For example, if gamma has a value of 0.5, the contribution of the self-concept and learning based on PEs to the final estimation is symmetrical. Note that *P^m^* represents accumulated feedback-based learning.


$$P^{m}\left(t+1\right)=E(t)\;+\sum_{i=2}^{t-1}\alpha \;\cdot PE(i)\;\cdot SIM(i,t+1)$$

$$P(t)=S(t)\;\cdot \gamma \;+(1-\gamma )\;\cdot P^{m}(t).$$

#### 2.2.5. Model 5: self-adjusted fine granularity (2 learning rates)

Model 5 combines model 4 with the dual learning rates from model 3.


$$P^{m}(t+1)=E(t)+\sum_{i=2}^{t-1}\alpha \left\{+,-\right\}\cdot \;PE(i)\;\cdot SIM(i,t+1)$$

$$P(t)=S(t)\;\cdot \gamma +(1-\gamma )\;\cdot P^{m}(t).$$

### 2.3. Model fit and comparison

We fitted and compared our computational models within the hierarchical Bayesian inference (HBI) framework. This framework has garnered popularity given its robustness and higher accuracy in parameter estimation and model selection in comparison to other fixed-effect approaches [58]. HBI offers several advantages for concurrent parameter estimation and model comparison by accounting for the hierarchical structure of the data and treating model identity as a random effect. That is, for each subject, rather than assuming that all models describe all subjects uniformly (a fixed effect), HBI considers that each subject could be described by any model within a set of candidate models. When treating model identity as a random effect, the model identity for each participant is represented as a multinomial variable. This multinomial variable represents the probabilities associated with each candidate model being the ‘true’ generator of that participant’s observed data. This procedure has proven to make model comparison less susceptible to outliers [58]. HBI implements a hierarchical approach that estimates the population distribution over the model parameters and the parameters of each individual subject, given the population distribution, regularizing individual parameter estimates. HBI method for model comparison includes estimating the probability of each individual from being generated by each model and utilizes it to weight the effect of individual datasets into model fit. It also allows for computing robust metrics for model selection, such as the protected exceedance probability (PXP), which represents the probability that each model is the most likely across all individuals, accounting for the possibility that differences in model evidence are due to chance [58,59]. We fitted our models using the computational and behavioural modelling toolbox (https://payampiray.github.io/cbm) implemented in Matlab (V. 2021, a). All models were fitted employing wide Gaussian priors [58].

#### 2.3.1. Parameter recovery

To assess the robustness of our computational models, we performed a parameter recovery analysis. Parameter recovery is a technique designed to evaluate the extent to which a computational model accurately estimates (or recovers) known parameter values. Specifically, we simulated 200 datasets using randomly drawn parameters, adding noise in the final step of simulation. In brief, parameter recovery tests the ability of a model to capture the true underlying processes generating the data (the known simulated parameters) through the parameters it estimates after fitting (the recovered parameters). The correlations for parameters within our best-performing model (model 4) were robust: learning rate (*r* = 0.948), gamma (*r* = 0.996). Correlations between simulated and recovered parameters for all models are presented in the electronic supplementary material, figure S3. Data were also simulated 200 times for the construction of a confusion matrix, which assessed the distinguishability of our models. That is, each model’s capacity to be accurately identified from the data it generated. We employed exceedance probability to quantify the likelihood that each model was the best fit for the data it had generated, as well as for the data generated by other models. Results from this analysis yielded an identity matrix, where all exceedance probabilities for the data-generating models hit 1, with all others at 0, demonstrating robust model distinguishability.

For all studies reported in this research, data and analysis code are available at https://osf.io/mkj9p/?view_only=6d5a07a79d7f420d818733cf5f636b14. Data were analysed using Matlab (V. 2021, a).

### 2.4. Results

Before the analysis based on the computational models introduced above, we conducted a more general test aimed at investigating whether participants were learning during the task. We modelled absolute PEs as a function of time (i.e. trial number) by means of a generalized additive model (GAM), a flexible version of linear regression that allows capturing nonlinear relationships by using smooth functions instead of straight lines [60]. The results of this analysis indicated a significant effect of time on PEs (*p* < 0.001), indicating a decrease in PEs in both groups throughout the task (electronic supplementary material, figure S2). Next, to gain a clearer, model‐free picture of how much participants’ own self‐views influenced role estimates, we ran a linear mixed‐effects analysis predicting each trial’s role rating from the participant’s self‐rating, the role‐relevance condition (self-relevant versus non-self-relevant) and their interaction. Crucially, the self-rating × role-relevance interaction was significant (*β* = 0.169, SE = 0.019, *t* = 8.741, *p* < 0.001), confirming that self-ratings exerted a stronger influence on role estimates when the role was self-relevant (*β*_self-relevant_= 0.292, SE = 0.013, 95% CI (0.266, 0.318)) than when it was not (*β*_non-self-relevant_ = 0.123, SE = 0.014, 95% CI (0.094, 0.151)). We also calculated, for each participant, the Pearson correlation between their self-ratings and their role ratings across trials. These correlations were higher in the self-relevant groups (*M* = 0.438, s.d. = 0.219, 95 % CI (0.382, 0.494)) than in the non-self-relevant groups (*M* = 0.158, s.d. = 0.204, 95 % CI (0.105, 0.211); a difference that was statistically significant: *t*(119) = 7.241, *p* < 0.001, *d* = 1.32).

To determine which computational model best captured participants’ responses, we conducted model fitting and comparison employing HBI, which estimates individual parameters hierarchically while comparing candidate models [58]. HBI integrates the benefits of hierarchical modelling for parameter estimation with the strengths of methods that consider model identity as a random effect (see §2.1 for details).

Results for the group that learned about a self-relevant role (that is, first-grade psychology students learning about the profile of a ‘well-adapted psychology student’) indicated that model 4 (self-adjusted granularity model) was the best-fitting model (model frequency: 89.72%). We computed the PXP, which assesses the probability that a given model is more commonly expressed than any other competing model in the model space while accounting for the null possibility that differences in model evidence are due to chance [58,59]. Results from PXP analysis corroborated model 4 as the winning model (PXP = 1; electronic supplementary material, figure S3). Model 4 accounts for how an individual’s self-concept can modulate the impact of external feedback on their learning and adjustment of estimations about role-representative attributes. Model 4 integrates self-ratings with predictions informed by fine granularity learning, underscoring the importance of the self-concept in shaping the learning process.

We conducted the same analysis for the group learning about a non-self-relevant role. HBI results for computational model comparison indicated that the winning model was also model 4 (self-adjusted granularity model) (model frequency: 97.33%). Results from PXP analysis corroborated model 4 as the winning model (PXP = 1; electronic supplementary material, figure S3). The consistent success of model 4 across both groups underscores its robustness as a framework for learning about different roles. This recurrence might indicate that the core mechanism of balancing feedback-informed learning with self-evaluations is applicable regardless of the role’s relevance to the learners.

**Figure 2.**
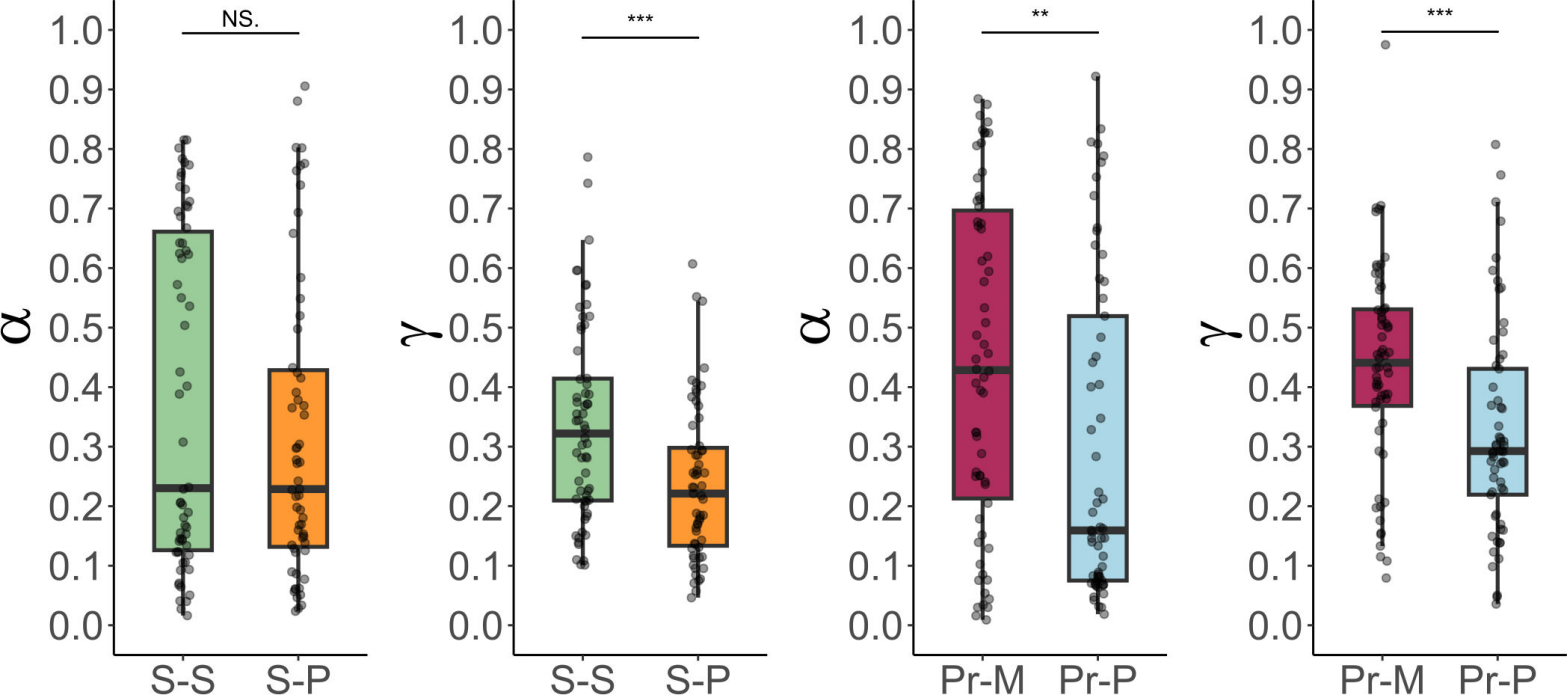
Comparative analysis of computational parameters *α* (learning rate) and *γ* (weight of self-concept) across study groups. (Left) Participant 1’s data from Study 1, with college students (*s*) assessing the role of a college student (S-S, group 1, green) or a police officer (S-P, group 2, orange). (Right) Participant’s data from Study 2 involving pregnant women (Pr) evaluating the mother role (Pr-M, group 1, maroon) or police officer role (Pr-P, group 2, light blue). Both sections illustrate boxplots of participants’ estimations regarding a ‘well-adapted [role]’ with *α* and *γ* parameters displayed on the left and right, respectively, for each subgroup. Jittered points represent individual data.

Given the recurrence of model 4 (self-adjusted granularity model) as the winning model in both groups, we next explored potential distinctions in the dynamics of learning by comparing its computational parameters—learning rate (*α*) and gamma (*γ*)—between participants learning about self-relevant and non-self-relevant roles. This analysis aimed to discern how the relevance of the role to participants’ anticipated life situations influenced their integration of new role-related information. We hypothesized that learning about self-relevant roles involved a stronger reliance on pre-existing self-concepts (indicated by a higher gamma), thereby controlling the dissonance between current self-views and the anticipated demands of the new roles. Results indicated that participants learning about a self-relevant role displayed a higher gamma parameter compared to those who learned about a non-relevant role (self-relevant: *M* = 0.336, s.d. = 0.134; non-self-relevant: *M* = 0.237, s.d. = 0.130, *t*(115.32)= 3.681, *p* < 0.001, *d* = 0.685, 90% CI (0.369, 0.999)). No differences were found for the learning rate (self-relevant: *M* = 0.377, s.d. = 0.281; non-self-relevant: *M* = 0.318, s.d. = 0.251, *t*(118.59)= 1.233, *p* = 0.219, *d* = 0.226, 90% CI (−0.076, 0.529); figure 2). The increased gamma parameter in those participants learning about self-relevant roles indicates a stronger reliance on self-evaluations in shaping their perceptions of role-specific traits. This heightened use of self-concept suggests that individuals are more inclined to utilize current self-views to modulate the impact of external feedback on the learning process when the role was seen as directly pertinent to their current or anticipated social contexts.

## 3. Experiment 2

### 3.1. Methods

#### 3.1.1. Participants

For this study, we recruited pregnant women by means of the online from the Prolific platform (https://www.prolific.com/) and were compensated with 5.28€ per participation.

As in experiment 1, experiment 2 involved two groups, each comprising 65 pregnant women without prior childbirth experience. Participants engaged in learning about roles that were either directly relevant to them (‘Mother’) or not (‘Police Officer’). The average age of the group focusing on the self-relevant ‘Mother’ role was 30.67 years (s.d. = 2.97), while the group assigned the ‘Police Officer’ role had an average age of 29.77 years (s.d. = 3.28). Average months of gestation were 5.71 (s.d. = 3.22) for group 1 (Mother role) and 6.08 (s.d. = 2.51) for group 2 (Police Officer role). Due to incomplete participation, missing more than 20% of the trials, three participants from the ‘Mother’ group and two from the ‘Police Officer’ group were excluded from the experimental sample. Two extra participants from the ‘Police Officer’ group were excluded due to giving the exact same rating in more than 80% of the trials during the experimental task. The final samples were composed of 62 and 61 participants, respectively. This set-up aimed to test the generalizability of our findings in experiment 1 to other populations undergoing important life transitions with strong societal expectations [15,43].

#### 3.1.2. Procedure

Similar to experiment 1, participants learned about traits defining either a well-adapted mother or a well-adapted police officer. They provided self-ratings and evaluated traits deemed necessary for these roles, while receiving feedback based on evaluations from external groups. For the ‘mother’ role, feedback was sourced from experienced mothers, while the ‘police officer’ feedback was the same used in experiment 1.

### 3.2. Results

Mirroring experiment 1, results from the general test of the effect of time on PEs (GAM) indicated a negative-like relationship between time and PEs in both groups (Pregnant-Mother: *p* = 0.019, Pregnant-Police officer: *p* = 0.008; electronic supplementary material, figure S2), indicating a reduction in PEs throughout the task. As in the previous experiment, we ran a linear mixed‐effects model predicting each trial’s role rating from the participant’s self‐rating, the role‐relevance condition (self-relevant versus non-self-relevant) and their interaction. Crucially, the self-rating × role-relevance interaction was significant (*β* = 0.079, SE = 0.021, *t* = 3.877, *p* < 0.001), confirming that self-ratings exerted a stronger influence on role estimates when the role was self-relevant (*β*_self-relevant_ = 0.473, SE = 0.014, 95% CI (0.445, 0.502)) than when it was not (*β*_non-self-relevant_ = 0.394, SE = 0.014, 95% CI (0.365, 0.442)). We also calculated, for each participant, the Pearson correlation between their self-ratings and their role ratings across trials. These correlations were higher in the self-relevant groups (*M* = 0.588, s.d. = 0.179, 95 % CI (0.543, 0.634)) than in the non-self-relevant groups (*M* = 0.394, s.d. = 0.224, 95 % CI (0.336, 0.451); a difference that was statistically significant: *t*(121) = 5.311, *p* < 0.001, *d* = 0.96).

As for the analysis based on computational models, HBI results indicated that the self-adjusted granularity model (model 4) was the winning model (Pregnant-Mother: model frequency: 78.1 %, PXP = 1, Pregnant-Police officer: model frequency: 98.46%, PXP = 1; electronic supplementary material, figure S3), confirming its robustness across different roles and populations.

As in experiment 1, we next compared computational parameters between groups (Pregnant-Mother versus Pregnant-Police officer). Participants learning about a self-relevant role showed both a higher learning rate (self-relevant: *M* = 0.441, s.d. = 0.280; non-self-relevant: *M* = 0.298, s.d. = 0.271, *t*(120.97)= 2.857, *p* = 0.005, *d* = 0.519, 90% CI (0.214, 0.822)) and a higher gamma parameter (self-relevant: *M* = 0.433, s.d. = 0.172; non-self-relevant: *M* = 0.325, s.d. = 0.177, *t*(120.78) = 3.411, *p* < 0.001, *d* = 0.635, 90% CI (0.320, 0.947)) than those learning about the ‘police officer’ role (figure 2B).

#### 3.2.1. Control analysis for experiments 1 and 2

Although our primary analyses already revealed that participants learning about self-relevant roles exhibited a higher gamma compared to those learning about non-self-relevant roles, it might be possible that these differences arise from the fact that leveraging their self-concept might have been slightly more effective at adapting to feedback in the self-relevant groups. That is, these differences may reflect a more optimal learning strategy within model 4.

To explore this possibility, we compared the deviation of empirical parameters from optimal parameter values—derived by fitting the model to directly predict feedback ratings [37] (i.e. by optimizing parameters for ‘learning’ rather than for fitting participants’ actual ratings)—as a function of role relevance by using a 2 (parameter type: empirical versus optimal) × 2 (role relevance: self-relevant versus non-self-relevant) mixed ANOVA.

For the learning rate, we did not find any significant interaction (students: *F*_1,119_ = 0.548, *p* = 0.461, *η²* < 0.001, 90% CI (0, 0.045), pregnant women: *F*_1,121_ = 0.934, *p* = 0.335, *η²* < 0.001), 90% CI (0, 0.053)), suggesting that although there was an apparent higher sensitivity to PEs in the self-relevant (versus non-self-relevant) learning group of pregnant women (figure 2), the deviation of the learning rate from its optimal value did not differ between conditions. In contrast, for the gamma parameter, a significant interaction emerged in both students and pregnant women cohorts (students: *F*_1,119_ = 14.077, *p* < 0.001, *η²* = 0.105, 90% CI (0.033, 0.204), pregnant women: *F*_1,121_ = 11.499, *p* < 0.001, *η²* = 0.086, 90% CI (0.023, 0.181)). Post hoc comparisons suggested that the difference between empirical and optimal gamma values (gamma empirical–gamma optimal) was greater for participants in the self-relevant role condition (students self-relevant vs non-self-relevant: *M*_diff_ = 0.101, SE = 0.026, *t*(119) = 3.752, *p* < 0.001, *d* = 0.687, 90% CI (0.687, 0.996), pregnant women self-relevant vs non-self-relevant: *M*_diff_ = 0.107, SE = 0.031, *t*(121) = 3.391, *p* < 0.001, *d* = 0.616, 90% CI (0.309, 0.921); electronic supplementary material, figure S5). These results suggest that participants learning about self-relevant roles deviated more from the optimal parameter solution by incorporating a higher gamma value, consistent with a strategy aimed at reducing dissonance between role estimations and self-views. This stronger deviation from optimal gamma values suggests that participants employ a biased strategy consistent with motivated reasoning [7,19,21,40,46,55,61] to preserve self-concept integrity when learning about their upcoming social roles. This bias appears to be a mechanism to minimize self-role dissonance, even at the cost of reduced optimality in feedback learning.

Next, we aimed to directly assess whether the effect of self-relevance on our key model parameters generalizes across distinct life transitions. We conducted 2 (cohort: students, mothers) × 2 (role relevance: self-relevant, non-self-relevant) between-subjects ANOVAs on both the learning rate (*α*) and self-weight (*γ*) parameters. This analysis was motivated by the idea that generalizability is best established if the relative increase in *α* and *γ* for self-relevant roles does not significantly differ across cohorts (i.e. a non-significant interaction).

For learning rate (*α*), there was a significant main effect of role relevance, (*F*_1, 240_ = 8.427, *p* = 0.004, *η²* = 0.034, 90% CI (0.006, 0.081)), with higher α for self-relevant roles (*M* = 0.409, SE = 0.024, 95% CI (0.361, 0.457)) than non-self-relevant roles (*M* = 0.308, SE = 0.024, 95% CI (0.259, 0.357)). The main effect of cohort was not significant, (*F*_1, 240_ = 0.375, *p* = 0.541, *η²* = 0.002, 90% CI (0, 0.021)), students: *M* = 0.348, SE = 0.024, 95% CI (0.229, 0.397), pregnant: *M* = 0.369, SE = 0.024, 95% CI (0.321, 0.418)), and the cohort × role-relevance interaction was not significant (*F*_1, 240_ = 1.405, *p* = 0.237, *η²* = 0.006, 90% CI (0, 0.032); see figure 2, for the marginal means).

For self-weight (*γ*), there were significant main effects of role relevance, (*F*_1, 240_ = 24.660, *p* < 0.001, *η²* = 0.093, 90% CI (0.042, 0.158), self-relevant: *M* = 0.385, SE = 0.014, 95% CI (0.356, 0.414), non-self-relevant: *M* = 0.282, SE = 0.014, 95% CI (0.252, 0.311)) and cohort (*F*_1, 240_ = 19.739, *p* < 0.001, *η²* = 0.075, 90% CI (0.029, 0.135), students: *M* = 0.287, SE = 0.014, 95% CI (0.258, 0.316), pregnant: *M* = 0.380, SE = 0.014, 95% CI (0.351, 0.409)). No significant interaction was found (*F*_1, 240_ = 0.044, *p* = 0.832, *η²* < 0.001, 90% CI (0, 0.010)).

Finally, although model 4 consistently outperformed the other models, we aimed to inspect whether it truly recapitulates participants’ behavioural patterns. To that end, we conducted group-level posterior-predictive checks for each of our four groups. For each dataset, we drew parameters from the posterior of model 4 and simulated new ‘Role Estimation’ trajectories trial by trial. We then compared the 95% credible intervals of those model-generated trajectories against the observed mean trajectory and the model’s own posterior-predictive mean. Across all four groups, the empirical data lie within the model’s credible bands, and the posterior-predictive mean closely tracks the observed time course in early, middle and late trials alike. Moreover, model 4 reproduces both the absolute level and the trial-to-trial fluctuations of participants’ estimates without systematic bias or under-/over-dispersion (electronic supplementary material, figure S6).

## 4. Discussion

In this research, we studied how individuals learn about upcoming social roles from the onset of two significant life transitions: becoming a university student and becoming a mother. Our findings reveal that individuals employ complex computational strategies that align role-related information with their existing self-concept. Notably, we found that when learning about roles directly relevant to their upcoming social contexts, individuals’ self-concept plays a stronger role in modulating their learning process, which mitigates potential dissonances between the anticipated demands of their new roles and their current self-views. By investigating role learning from a computational perspective, we shed light on how individuals might start reconciling the delicate trade-off between stability and adaptation from the onset of significant life transitions.

We found that across all study groups, participants’ self-concept consistently modulated learning based on PEs. The consistency of this strategy across varied roles and populations highlights its potential as a generalized model for role learning. These findings extend recent computational models focused on learning personality traits and preferences of single individuals [37,38] by including the fundamental role of the self-concept as a latent structure capable of modulating social learning processes. Importantly, the comparative analysis of computational parameters indicated that individuals learning about their forthcoming roles (versus non-self-relevant roles) displayed a stronger modulatory influence of their self-concept in the learning process. These findings are consistent with the notion that preserving self-concept stability is instrumental to our well-being [1,6,10,19,21].

The recurrence of the enhanced modulatory effect of the self-concept among participants learning about self-relevant roles might be promising for understanding how individuals reconcile stability and adaptation from the onset of life transitions. Specifically, participants learning about self-relevant roles might have avoided self-role dissonance by combining the modulatory effect of the self-concept with asymmetric learning dynamics, that is, dual learning rates. Dual learning rates were based on whether feedback increased or decreased the dissonance between participants’ self-views and role expectations. However, we did not find evidence supporting an asymmetric learning strategy. Participants’ preference for a self-adjusted model with a single learning rate suggests that they may prioritize a strategy that allows updating their role-related perceptions cohesively over one that requires recalibrating learning rates based on the feedback’s relation to their current self-role dissonance.

Cohesive yet self-adjusted role learning might offer an effective trade-off for individuals acquiring novel characteristics. However, previous findings suggest that individuals with low self-concept stability may resist expanding their identities to encompass new role characteristics [62]. In these instances, individuals might intensify their strategies for self-concept maintenance, potentially including a biased sensitivity to role-related information. Indeed, reduced self-concept clarity has been associated with psychopathological conditions and suboptimal role adaptation [11,63]. Future studies should extend the application of our computational models to these populations to further explore the generalizability of our findings.

Interestingly, we found that even when participants were learning about a role they did not personally aspire to (e.g. police officer), they still employed the same self-adjusted learning mechanism. This consistent usage of the self-concept—despite the role’s lack of personal relevance—suggests that whenever a social role carries strong cultural or societal expectations, learners may invoke the same strategies, potentially to keep new information aligned with their existing self-views, consistent with motivated reasoning. Alternatively, it might reflect an anchoring-and-adjustment process similar to that observed in social inferences about others [26,64]. As mentioned, differences between self-relevant and non-self-relevant role learning did not involve model identity but model expression (higher shrinkage of role estimation toward the self in the self-relevant groups). These differences are unlikely to be attributable to an anchoring under uncertainty process [26,65], since in those instances, one would expect higher gamma values in the non-self-relevant (i.e. potentially less familiar) roles. Instead, our results suggest the opposite: *γ* is considerably larger when the role directly implicates the self. This finding aligns with prior work suggesting that individuals strive to strategically manage dissonances between current and desired or optimal self-states to preserve self-concept integrity and mental health [7,41].

We also found that pregnant women exhibited a higher gamma parameter than college students. Although this effect was not the primary focus of our study, it is consistent with prior research indicating that the clarity and stability of the self-concept tend to increase from late adolescence through adulthood [66,67], which might help individuals in using their self-concept against the pure incorporation of social information [7]. These findings suggest that incorporating the self-concept into computational models of social learning is crucial, as it might reflect the inherent influence of an individual’s identity on processing and responding to social information. Future research should extend our findings by incorporating roles differing in other dimensions apart from self-relevance, including social relevance or role alignment with social or personal values.

Here, we elucidated the strategies individuals may utilize to balance role learning with self-concept preservation from the onset of important life transitions. These findings may carry important practical implications. Life transitions have been associated with disruptions in self-concept integrity [68,69], a well-established predictor of psychological functioning and well-being [1]. In turn, lack of identification with new social roles has been related to negative outcomes; for example, mothers struggling to identify with their new maternal role experience impairments in socio-emotional functioning [15]. Therefore, delineating role-learning dynamics may be crucial for understanding the well-being correlates of individuals undergoing life transitions [70]. The utilization of computational parameters as markers of role-learning strategies might be promising for predicting individual trajectories of role adaptation and self-concept preservation. If this notion holds true, this might open the door to novel preventive and interventional strategies informed by computational evidence.

The computational models presented here can be extended to shed light on other complex phenomena in the context of role transitions; for example, investigating how individuals adapt to and overcome unexpected and potentially threatening roles, such as those imposed by severe medical conditions. There is evidence that individuals diagnosed with severe medical conditions, such as cancer, undergo profound transformations related to their health condition and restrictions in their lives [71]. Moreover, advancements in medicine have led to a growing population of cancer survivors who need to readapt to daily life and redefine their ‘cancer patient’ identities [72]. This is a complex process that varies among individuals and can have an important impact on healthcare usage and psychosocial distress. Our computational models could serve as a valuable tool for tracking and predicting individual trajectories of both adaptation to and exit from the cancer patient identity, potentially informing current healthcare practices.

The current research also refines the understanding of the computational principles of social learning. Advancements in this field have recently unveiled that computational models can capture individuals’ learning about complex social knowledge structures [37,38]. Here, we have shown that when the learning process is self-relevant, individuals’ self-concept acts as a stronger modulatory mechanism for learning about reference groups. These findings might have direct implications for understanding how people learn about the personality of single individuals. We anticipate that when anticipating life transitions, individuals learning about single persons within their forthcoming social groups will employ similar strategies. Aligning these individuals’ characteristics with their own self-concept might increase their subjective similarity to group members and bolster a sense of group belongingness [73]. Similarly, our findings complement and extend existing computational work by showing that the self not only informs social learning [74,75] but also actively modulates the mapping between self-concept and role-specific trait demands. By conceptualizing role learning as trait’s adaptivity estimation, we reveal that individuals employ their self-concept as a modulatory force that helps reconcile adaptive demands of upcoming social roles with existing identities, thereby extending the scope of prior computational work in social cognition.

Finally, our findings extend other existing models of self-related social learning and highlight the opportunity for integration with recent work. Recent studies in this area have demonstrated that when targeting individuals’ self-concepts with social feedback, not all self-beliefs are equally malleable [28,31]. Rather, their malleability is related to their structural importance or ‘centrality’ within the broader network of self-knowledge. In these works, the authors assessed centrality based on subjective estimates of causal relationships among self-traits. Their results indicated that highly central traits tend to be less susceptible to change when confronted with social feedback. However, in those studies, centrality was treated as a feature for interpreting results but was not directly embedded in their computational models. In contrast, our approach explicitly incorporates the interconnectedness among traits into the core equations of the learning process, modelling how feedback about one trait can influence beliefs about related traits through their empirical correlations. Integrating these approaches offers a clear path for future research. Computational models could be further refined by explicitly including trait centrality as a moderator of feedback propagation. This would allow for nuanced predictions about when and how particular estimations are more or less likely to change in response to social input.

## 5. Limitations

Participants in both experiments were healthy adults undergoing specific life transitions: first-year university students transitioning into a new academic role and pregnant women transitioning into motherhood. Thus, our findings specifically target individuals experiencing normative, anticipated life transitions with clearly defined forthcoming social roles. Future research is needed to assess the robustness of these findings in diverse populations, including those facing atypical or involuntary transitions. Moreover, we found similar patterns across both cohorts (students and pregnant women); however, the specific analysis conducted on computational parameters as a direct comparison between groups (see control analysis) needs to be interpreted with caution, since it is based on a null effect (lack of statistical significance of the interaction cohort × role relevance). Our framework primarily addressed learning about personality characteristics. Although those characteristics are considered summary representations of behaviour, feelings and cognitive processes, future research could be enriched by investigating how individuals learn about other role-relevant aspects, such as values or expected behaviours. Such generalization would solidify the utility of our computational models for understanding role transitions and social learning more broadly. Finally, in the present work, we treated the drive to learn about future environments and the motivation to avoid self-role dissonances as fundamental motives based on prior research. However, we did not directly measure participants’ subjective motivation to align with feedback or to preserve their self‐views. Although our non‐self‐relevant role-learning condition offers evidence that self‐relevance modulates self‐weighting, we did not experimentally manipulate participants’ need for adaptation or for self‐stability. Future studies should incorporate explicit motivational assays (e.g. self‐report scales, incentive manipulations) to directly test whether the up‐weighting of self‐concept in self‐relevant conditions arises from strategic, motivational processes.

## Data Availability

For all studies reported in this research, data and analysis code are available at [76]. Supplementary material is available online [77].
